# Medical Service Afloat

**Published:** 1924-09

**Authors:** 


### MEDICAL SERVICE AFLOAT.

### A CAREER FOR THE NEWLY QUALIFIED.

It is a little surprising that the life offered to officers of the Royal Naval Medical Service does not appeal more generally to recently qualified men. To be in the Navy is certainly to see the world in most congenial company, and the position of the doctor is certainly a pleasant one, and, on the more material side, it can at least be said that the pay is good and the pensions commensurate with it. Nor is this all. There are many real opportunities for scientific work, and first-class original research is undoubtedly rewarded by promotion on grounds of professional merit. Apart from such special promotion a surgeon can achieve the rank of Surgeon Lieut.-Commander in six years from his date of entry, and in the same way another six years brings him to the period for promotion to Surgeon-Commander. Promotions to Surgeon-Captain, and later to Surgeon-Rear-Admiral, are made by personal selection and confined to officers fitted in both professional and administrative ability for these ranks.

### Opportunities for Skill and Personality.

It is sometimes said that the Naval Medical Service gives a man little or no opportunity to exercise his skill and personality, but it is only to the most unobservant that matters can so appear. The extraordinary conditions under which much of his work is carried out furnish perpetual surprises and problems for the medical man afloat. It is undeniable that the influence of the Medical Officer must always be felt by both officers and men, and in such conditions there is no excuse for absence of practical interest in the day's work and its complexities. Many of the changes in naval life at sea made during the last few years have arisen directly from research instituted or carried through by the Medical Service. The science of nutrition, adaptation to tropical and other climes, physical training and ophthalmology are only a few instances of the many branches open to the progressive observer. One recently introduced advantage available to the newly-qualified man is the adoption of short service schemes, whereby a Surgeon-Lieutenant after six months' service under naval conditions can apply to be transferred to the permanent list. The details of rates of pay, etc., cannot be given here, but suffice it again to say that they are, for the medical profession, relatively good. There is a Naval Dental Service which offers similar opportunities to qualified dental surgeons, except that ranks higher than Surgeon-Commander (D) are not open to them.

### The Merchant Service.

There is, of course, an alternative method of service afloat—namely, that obtained in the various passenger and merchant services. Conditions vary so much with different companies and routes that no concise description of the possibilities can be given. Most of the larger companies have their regular medical staff, entry into and progress in these professional channels differing in kind only from those of the Naval Service. With regular appointments and promotion it is by no means as easy as it was to obtain medical posts in the bigger ships, but to those who simply need an enjoyable and yet not altogether unproductive holiday, a few trips with the less well-known passenger lines are well worth while. Several of these companies regularly apply to the medical schools for temporary medical service, and thus the Dean of a man's own school is frequently able to put him in touch with the line concerned. Otherwise it is not a difficult matter to obtain introduction to the companies, and a man seeking a temporary appointment has usually little difficulty in obtaining a post. Here the word " temporary " should again be emphasized, because, if permanent service with the sea and its ships is attractive, there is certainly no better way of obtaining it than joining the Naval Service. Good permanent work with the civil lines is far more difficult to find, and, when found, of very doubtful advantage over that offered by the Senior Service.

